# Prevalence and Risk Factors of HIV, Syphilis, Hepatitis B and C Among Female Prisoners in Isfahan, Iran

**DOI:** 10.5812/hepatmon.6144

**Published:** 2012-07-30

**Authors:** Zary Nokhodian, Mohammad Reza Yazdani, Majid Yaran, Parisa Shoaei, Mina Mirian, Behrooz Ataei, Anahita Babak, Mehdi Ataie

**Affiliations:** 1 Infectious Diseases Research Center, Isfahan University of Medical Sciences, Isfahan, IR Iran; 2 Nosocomial Infection Research Center, Isfahan University of Medical Sciences, Isfahan, IR Iran; 3 Isfahan Pharmaceutical Sciences Research Center, Isfahan, IR Iran; 4 School of Medicine, Najafabad Branch, Islamic Azad University, Isfahan, IR Iran

**Keywords:** HIV, Hepatitis B Virus, Hepatitis C, Syphilis, Prevalence, Risk Factors

## Abstract

**Background:**

Female prisoners are at risk of acquiring sexually transmitted infections (STIs). There has been no previous study regarding the epidemiological status of STIs among female prisoners in Isfahan, central Iran.

**Objectives:**

The aim of this study was to investigate the prevalence and risk factors of the aforementioned infections among women incarcerated in the central prison, Isfahan, to determine appropriate prevention measures.

**Patients and Methods:**

In a cross-sectional study, all of the 163 women incarcerated in the central prison, Isfahan in 2009, were voluntarily enrolled by the census method. After completing a checklist consisting of demographic, social, and risk factors, a 5ml blood sample was taken from each individual. The sera were analyzed for markers of the hepatitis B virus (HBV; HBsAg, HBsAb, HBcAb), hepatitis C virus (HCV; HCV antibodies), human immunodeficiency virus (HIV; HIV antibodies), and syphilis (RPR). Confirmatory tests were performed on HCV antibody-positive cases.

**Results:**

The mean age of the participants in the study was 34.54 ± 11.2 years old, 94.3% of these women were Iranian, and many of them had only a primary level of education. The prevalence of HBsAg, HBcAb, HBsAb, and HCV antibodies were; 1.2%, 7.4%, 12.9% and 7.4% respectively. No positive RPR or HIV antibodies were detected.

**Conclusions:**

A significant relationship was seen between the HCV antibody, drug injection and illegal sex in the women, and also between HBc-Ab and drug injection. Regular screening, educational programs, and facilitation of access to suitable treatment care should be widely implemented in the prison population. Testing for immunity against HBV should be considered on admission, and afterwards vaccination of all prisoners and an appropriate preventative approach should be applied.

## 1. Background

In many countries prisoners have a higher prevalence of blood-borne diseases and sexually transmitted infections (STIs) [1], this rate is also greater among adolescents compared to the general population [2]. Prisoners are at risk of acquiring; human immunodeficiency virus (HIV), hepatitis B and C virus (HBV and HCV) infections due to their lifestyles and high risk behavior, that includes; illicit drug injection use inside or outside the prison, unsafe sex, multiple sexual partners, homosexuality and tattooing [3]. In addition, prison inmates are affected by; prevailing social health problems, illegal behavior, and limited educational opportunities [4]. The seroprevalence of hepatitis B surface antigens (HBsAg), antibodies against hepatitis B core antigens (HBcAb), and HCV antibodies have been reported to be up to 42.9% [1], 46.03% [5], and 53% [3] respectively in prison inmates. The increasing incidence of HIV infections and acquired immunodeficiency syndrome (AIDS) among prisoners in a number of countries has further complicated this issue. HIV prevalence has been reported in up to 15.1% of this population as well [1]. Furthermore, several studies in a variety of HIV-positive populations have shown a high incidence of syphilis in prison populations [6][7], and some studies have postulated that HIV is a risk factor for syphilis [8][9]. Both diseases are associated with a history of incarceration [8][10]. It is estimated by the Centers for Disease Control and Prevention (CDC, Atlanta, USA ), that approximately eight million prisoners return to their communities annually and that may be dangerous for community health. A recent statement by the World Health Organization (WHO) concerning the health of prisoners, considers that that transmission of infectious diseases in prisons can be controlled with proper strategies [11]. In addition, women are susceptible to STIs due to their specific anatomy , and the reported prevalence of these infections in incarcerated females is greater than for males, despite fewer numbers of female prisoners compared to males [12]. Because of their different life styles, the women might be exposed to; multiple sexual partners, unsafe sex, addiction, and various kinds of infections.

## 2. Objectives

There has been no previous study regarding the epidemiological status of HIV, syphilis, and hepatitis B and C virus infections among female prisoners in Isfahan, central Iran. Accurate data on the prevalence of such diseases are required in order to determine appropriate prevention measures to protect the community, and monitor patterns of health care and clinical interventions. Thus, the aim of this study was to investigate the prevalence and risk factors of HIV, syphilis, and hepatitis B and C virus infections by serological methods among women incarcerated in the central prison of Isfahan, Iran, in 2009.

## 3. Patients and Methods

This is a cross-sectional study approved by the Ethics Committee of the Isfahan University of Medial Sciences. In this study, all of the 163 women incarcerated in the central prison of Isfahan in 2009 were enrolled voluntarily by the census method after they had signed a written informed consent form. For each individual a checklist was recorded by a trained female social worker in the prison and enrollees could skip any question. The checklist included demographic and social data and high risk behaviors including; age, educational level, residence location, crime type, duration of imprisonment, number of times arrested, marital status, number of marriages, history of temporary marriage, history of high risk sex, how many times her husband had been married, history of husband’s imprisonment, use of illicit drugs, status of other family members’ drug addiction, and finally, the response rate of the invited cases. Regarding illicit drugs, use of some drugs with street names such as heroin, opium, hashish, crack, norjisac, tamjisak, and glass were considered. The routes of illicit drug consumption were divided into three categories including; inhalation, injection, and ingestion. Any individual who had injected even once was included in the injection group. Type of crime was divided into three subgroups including; criminal, penal, and retributive. Regarding the duration of imprisonment, if there had been a number of arrest times, then all periods of incarceration were added together. In order to detect HBV, HCV, HIV, and syphilis, a 5 ml blood sample was taken from each individual and sent to the Infectious Diseases and Tropical Medicine Research Center of Isfahan for separation of serum and testing. Samples were tested for markers of HBV (HBsAg, HBsAb, HBcAb), HCV (HCV antibody), and HIV (HIV antibodies), using the enzyme-linked immunosorbent assay (ELISA) method (Diapro, Italy) with more than 90% sensitivity. HCV antibody-positive samples were confirmed by Recombinant Immunoblot Assay (RIBA) (Diapro, Italy). Samples were also tested for syphilis, using the rapid plasma regain (RPR) method (Omega, Scotland) with more than 90% sensitivity. Data analysis was prepared using SPSS software, version 18, descriptive statistical and χ^2^ methods and P < 0.05 was considered to be statically significant.

## 4. Results

The response rate of the invited cases was complete. Mean age of the women participating in the study was 34.54 ± 11.2; the youngest and oldest were 15 and 70 years old respectively. Age distribution of persons participating in the study is shown in Table 1. This group included 94.3% Iranian, 4.4% Afghan, and 1.3% Arabian cases. 86.1% percent of these were from urban areas. Concerning temporary marriages, 29 inmates had a positive history, 125 inmates had a negative history, and nine patients did not answer definitely. These women were being held for various crimes; 49.7%, 27.7% and 22.6%, for penal, retributive and criminal crimes respectively. A history of incarceration in their husbands was seen in 44.3% of the cases. Multiplicity of marriage was reported in 32.8% of the women’s husbands in this study. Demographic data and arrest profiles are listed in Table 2. HBsAg, HBcAb, HBsAb and HCV antibodies were positive in 2 (1.2%), 12 (7.4%), 21 (12.9%) and 12 (7.4%) cases respectively. No positive cases of HIV or syphilis were observed. Two HBsAg-positive cases had positive HBcAb results that were compatible with chronic hepatitis B. Of the 21 HBsAb-positive cases, 10 (6.2%) cases had a positive HBcAb result that is compatible with a previously cured infection, and 11 (6.8%) cases had negative HBcAb results that were due to previous vaccination, remote infection or false positive results. Among the HCV antibody-positive cases, no simultaneous positive result of HBV markers was seen.

**Table 1:** Age Distribution of Persons Participating in the Study

**Age Groups, y**	**Patients, No. (%)**
15-24	27 (16.6)
25-30	39 (23.9)
30-35	29 (17.8)
36-45	32 (19.6)
45	36 (22.1)

**Table 2:** Bivariate Analysis of Risk Factors for Hepatitis B and C

	**Subjects , No. (%)**	**HBsAg+**^a^**, No. (%)**	**HBsAb+**^a^**, No. (%)**	**HBcAb+ **a**, No. (%)**	**HCV+**^a^**, No. (%)**
**Education**					
Illiterate	32 (20.3)	1 (3.1)	9 (28.1) b	6 (18.8)	2 (6.3)
Primary school	45 (28.5)	1 (2.2)	5 (11.1)	3 (6.7)	4 (8.9)
Guidance school	38 (24.1)	0	2 (5.3)	2 (5.3)	3 (7.9)
High school	15 (9.5)	0	3 (20)	1 (6.7)	0
Diploma and higher	28 (17.7)	0	2 (7.1)	0	3 (10.7)
**Marital status **					
Single	15 (9.5)	0	2 (13.3)	0	3 (20)
Married	80 (50.6)	2 (2.5)	10 (12.5)	5 (6.3)	5 (6.3)
Divorced	22 (13.9)	0	2 (9.1)	2 (9.1)	2 (9.1)
Widow	41 (25.9)	0	7 (17.1)	5 (12.2)	2 (4.9)
**Number of marriages **					
1 time	104 (73.2)	1 (1)	17 (16.3)	10 (9.6)	7 (6.7)
2 times	36 (25.4)	0	2 (5.6)	2 (5.6)	2 (5.6)
3 times and more	2 (1.4)	0	0	0	0
**Temporary marriage **					
Yes	29 (18.8)	0	2 (6.9)	2 (6.9)	2 (6.9)
No	125 (81.2)	2 (1.6)	19 (15.2)	10 (8)	9 (7.2)
**Vaginitis**					
Yes	13 (24.5)	0	3 (23.1)	2 (15.4)	0
No	40 (75.5)	0	9 (2.5)	4 (10)	5 (12.5)
**Number of arrests **					
1 time	120 (79.5)	2 (1.7)	14 (11.7)	8 (6.7)	9 (7.5)
2-3 times	28 (18.5)	0	5 (17.9)	2 (7.1)	2 (7.1)
4-5 times	3 (2)	0	1 (33.3)	1 (33.3)	1 (33.3)
**Contact with infectious patients**					
Yes	19 (12)	0	1 (5.3)	2 (10.5)	1 (5.3)
No	139 (88)	2 (1.4)	20 (14.4)	10 (7.2)	11 (7.9)
**Abortion**					
Yes	58 (36.7)	0	5 (8.6)	3 (5.2)	4 (6.9)
No	100 (63.3)	2 (2)	16 (16)	9 (9)	8 (8)
**Dialysis**					
Yes	1 (0.6)	0	0	0	0
No	157 (99.4)	2 (1.3)	21 (13.4)	12 (7.6)	12 (7.6)
**History of tattooing**					
Yes	57 (36.1)	0	9 (15.8)	5 (8.8)	7 (12.3)
No	101 (63.9)	2 (2)	12 (11.9)	7 (6.9)	5 (5)
**History of cupping**					
Yes	12 (7.6)	0	1 (8.3)	0	0
No	146 (92.4)	2 (1.4)	20 (13.7)	12 (8.2)	12 (8.2)
**History of ear piercing**					
Yes	158 (100)	2 (1.3)	21 (13.3)	12 (7.6)	12 (7.6)
No	0	0	0	0	0
**History of surgery**					
Yes	85 (53.8)	1 (1.2)	11 (12.9)	6 (7.1)	6 (7.1)
No	73 (46.2)	1 (1.4)	10 (13.7)	6 (8.2)	6 (8.2)
**History of blood transfusion**					
Yes	33 (20.9)	0	4 (12.1)	3 (9.1)	3 (9.1)
No	125 (79.1)	2 (1.6)	17 (13.6)	9 (7.2)	9 (7.2)
**History of dental work**					
Yes	131 (82.9)	2 (1.5)	15 (11.5)	9 (6.9)	9 (6.9)
No	27 (17.1)	0	6 (22.2)	3 (11.1)	3 (11.1)
**History of organ transplantation**					
Yes	1 (0.6)	0	0	0	0
No	157 (99.4)	2 (1.3)	21 (13.4)	12 (7.6)	12 (7.6)
**History of illegal sex **					
Yes	29 (18.4)	0	3 (10.3)	1 (3.4)	7 (24.1) c
No	129 (81.6)	2 (1.6)	18 (14)	11 (8.5)	5 (3.9)
**Addiction **					
Yes	49 (31)	0	9 (18.4)	6 (12.2)	10 (20.4) c
No	109 (69)	2 (1.8)	12 (11)	6 (5.5)	2 (1.8)
**Drug injection **					
Yes	5 (3.2)	0	2 (40)	2 (40) b	3 (60) c
No	153 (96.8)	2 (1.3)	19 (12.4)	10 (6.5)	9 (5.9)

^a^ Abbreviations: HBsAg, hepatitis B surface antigen; HBsAb, hepatitis B surface antibody; HBcAb, hepatitis B core antibody; HCV, hepatitis C virus

^b^ P value < 0.05

^c^ P value < 0.001

Forty nine imprisoned women and 39.2% of their family members had consumed illicit drugs. Route of consumption included injection in five persons, inhalation in 42 persons, ingestion in one person, and both inhalation and ingestion in one person. Twenty nine prisoners and 22.8% of their husbands (36 cases) had had multiple sexual partners. A significant statistical relationship was seen between the prevalence of HCV antibodies and a positive history of drug injection and illegal sex in the inmates, and also between HBcAb and drug injection (Table 2 and Table 3). No significant statistical relationship was seen between HBV or HCV infections and other risk factors.

**Table 3:** Multiple Logistic Regressions of Risk Factors for Hepatitis C Virus Antibody and Hepatitis B Core Antibody

	**Odds Ratio**	**95% CI**	**P value**
HCV-Ab a			
**Drug injection **		2.91-209.01	< 0.001
Yes	24.68		
No	1		
**History of illegal sex **		2.10-30.65	< 0.001
Yes	8.01		
No	1		
HBc-Ab a			
**Drug injection **		2.2-128.09	< 0.001
Yes	16.78		
No	1		
Age, y	1.06	1.01-1.12	< 0.05

^a^ Abbreviations: HCV-Ab, hepatitis C virus antibody; HBc-Ab, hepatitis B core antibody

## 5. Discussion

In this study, HIV antibodies and RPR tests were negative in all studied persons and that is compatible with similar studies conducted in Iran. The prevalence of HIV and HIV/syphilis in female prisoners was reported to be zero by Azarkar et al. [13] and Ghanbarzadeh et al. [14] respectively. Studies conducted in various other countries of the world show different results. In a study of seven prisons in Australia none of the incarcerated women were HIV-positive [3]. In a study in Pakistan one of the 84 incarcerated women had an HIV-1 infection [15]. In a multicenter study in Ghana [1] the prevalence of HIV and syphilis in female prisoners has been reported to be 15.1 and 36.1 respectively. In a prison in Brazil 13.9% and 22.8% of prisoners were positive for HIV and syphilis respectively [16]. In Portugal [17] and Italy [5] 10% and 5.6% of the female prisoners were HIV-positive, respectively. According to a study by Ghrari et al. [18], 23% of the female prisoners were infected with syphilis, and 2% were HIV-positive. It seems that despite the presence of addiction, illegal sex, illiteracy, and living in poor families, the low prevalence of syphilis and HIV found in Iran is most likely due to the low prevalence of these infections in the general community and the relatively low numbers of women studied in the prisons. In this study HBsAg, HBcAb, HBsAb and HCV antibodies were positive in; 1.2%, 7.4%, 12.9%, and 7.4% of cases respectively. In a study by Azarkar et al. [13], HBsAg and HCV antibodies were positive in 2.5% and 5.1% of female prisoners, respectively. The prevalence of HBsAg and HCV in female prisoners was reported to be; 7.5% and zero by Ghanbarzadeh [14] and Salehi [19], respectively. In Australia, the prevalence of HBcAb, HBsAb, HBsAg, and HCV antibodies were reported to be; 29%, 23%, 2%, and 53%, respectively [3]. The prevalence of HBcAb, HBsAg, and HCV antibodies in female prisoners in Italy was reported to be; 46.3%, 6.7%, and 20.6%, respectively [5]. In one study in a prison of Bologna in Italy, 433 prisoners which included 43 women, 8.1% were HBsAg-positive and 31.1% were HCV antibody-positive [20]. In research conducted in Ghana [1], the seroprevalence of HBsAg was reported to be 42.5% in the women. Rates of HCV antibodies in Brazil [16] and Portugal [17] were declared to be 16.2% and 11% respectively. In research conducted by Passadouro on 788 prisoners, including 89 women, the prevalence of HBsAg, HBsAb, HBcAb, and HCV antibodies, were reported to be; 3%, 40%, 40% and 42% respectively [21]. Discrepancies in the various study results may be due to differences in the prevalence of these infections in their communities. Results of this study (see Table 2- 3) indicate that there is a relationship between the prevalence of HCV antibodies and a positive history of drug injection and illegal sex in the prisoners, and also between HBc-Ab and drug injection. In all of the studies mentioned above [3][13][16][17][19][20], illicit drug injection use in the female prisoner or her sexual partner has been introduced as a risk factor and that may be due to the use of shared injection paraphernalia and high risk behaviors, that expose the individual to dangerous diseases such as AIDS and hepatitis viruses. The relative frequency of positivity of the various markers of HBV and HCV among female prisoners in Isfahan differs from the results of other similar studies in Iran and other parts of the world. This may be due to differences in the rate of infections in the various communities. Comparison of the results of this study with the prevalence of various markers of HBV among women in the general population in Isfahan were reported to be; 0.7%, 12%, and 4% for HBsAg, HBsAb, and HBcAb respectively [22]. These results indicate that Iranian prisons are also high risk places for infection transmission and this can have an effect on the rate of community infection producing a higher number of cases .

Despite the relatively low numbers in the study population and the limited number of positive cases that might complicate the determination of risk factors, we believe that this research represents a challenge to the health status of this group. Almost 85% of these women have no immunity against HBV, as well as this, the prison is not regarded as a site that is separate from the community. Many prisoners serve only short periods of imprisonment and a number of them return to their homes during their vacation or after completion of their imprisonment and they can transfer serious infectious agents into the community. Therefore, regular inmate screening, educational programs, focusing on prevention in order to promote low risk behaviors, and facilitating access to suitable treatment care, should be widely implemented as soon as possible for this population. In addition, testing for immunity against HBV should be considered on admission and after vaccination of all prisoners and an appropriate approach should be applied according to a vaccination schedule. This study also had several limitations. First, it included a relatively low number of subjects and a limited number of positive cases, and thus, it is difficult to make a definite statement about the risk factors. Second, serologic tests of HIV infection may produce false negatives in the initial weeks of acquiring an infection, and p24 antigen tests or nucleic acid tests are preferred during this period.
